# The association between Thoroughbred racehorse training practices and musculoskeletal injuries in Victoria, Australia

**DOI:** 10.3389/fvets.2023.1260554

**Published:** 2023-10-24

**Authors:** Adelene S. M. Wong, Ashleigh V. Morrice-West, Peta L. Hitchens, R. Chris Whitton

**Affiliations:** Equine Lameness and Imaging Centre, Melbourne Veterinary School, The University of Melbourne, Werribee, VIC, Australia

**Keywords:** Thoroughbred, racehorse, musculoskeletal, injury, training, racing, Australia

## Abstract

Catastrophic musculoskeletal injuries (CMI) in horses are associated with both too little and too much high-speed exercise. In order to advise trainers on training and management strategies that minimize the risk of musculoskeletal injury (MSI), a better understanding of how training practices affect MSI in racehorses is needed. Data from prospective studies relating training data and MSI are complicated by the gradual development of pathology and the effect of this on the ability of horses to train consistently prior to the identification of an injury. To circumvent this, 66 Australian Thoroughbred trainers were surveyed on their intended training practices, including rest, pre-training, and race-fit practices. Associations between intended training practices and catastrophic and non-catastrophic race day MSI outcomes in two-year-old and mature (≥three-year-old) horses were assessed using multivariable negative binomial regression models. The incidence of two-year-old race day MSI was lower for trainers who preferred shorter times (weeks) to trial, less time in fast work pre-trial (*p* = 0.003), shorter, more frequent rest periods (*p* < 0.01) and higher amounts of fast work at 15.5–16.7 m/s once race-fit (*p* = 0.001). The incidence of mature horse race day MSI was lower for trainers who preferred longer rest periods (*p* = 0.026) and a high-volume pre-trial training strategy comprising a high volume of slower speed gallop training and longer times to trial compared to fast and light training programs (*p* = 0.004) for their mature horses, in addition to higher amounts of fast work at 15.5–16.7 m/s for their race-fit two-year-olds (*p* = 0.012). Race day CMI incidence was lower for trainers who preferred lower volumes of fast gallop work for their race-fit mature horses (*p* < 0.05). These results suggest that two-year-old training practices could affect MSI risk later in a horse’s career, and that age and stage in training (pre-trial, race-fit) are important considerations when developing training practices to minimize the risk of MSI.

## 1. Introduction

Musculoskeletal injuries (MSI), the most common cause of fatalities in Thoroughbred racehorses, pose a significant welfare concern to both racehorses and their riders (1–3). Catastrophic musculoskeletal injuries (CMI) in horses are associated with both too little and too much high-speed exercise (4). Bone fatigue plays an important role in the development of MSI whereby the cyclic loading of bone during training and racing results in the accumulation of bone damage (5, 6). Excessive bone damage can occur when high speed training is introduced too rapidly in a poorly adapted skeleton. This situation may arise in young or inexperienced horses or following a period of rest. Excessive bone damage can also occur when training and racing continues for a period that exceeds the capacity of a well-adapted skeleton to recover from the accumulated fatigue. A training program that reduces the risk of fracture is therefore a program that minimises bone fatigue while introducing and maintaining the appropriate amount of high-speed exercise for bone adaptation and strength (5).

Studies in the US and UK have investigated the relationship between MSI and training workload based on training histories which could result in the inclusion of a forced reduction in workload due to pre-existing or underlying injury (7–10). This results in a potential confounding effect known as the ‘healthy horse effect’. This ‘healthy horse effect’ or survival bias describes the situation where fit and healthy horses are more likely to be training and racing but less likely to sustain an injury, therefore biasing the relationship between training histories and MSI (11). Furthermore, fatigue accumulation is not simply a function of speed and distance travelled by the horse, but also dependent on various other training-related factors including the frequency and duration of rest periods, as well as the rate at which high speed work is reintroduced (5, 12). The combination of these factors complicates the understanding of how racing and training histories affect injuries in racehorses.

Previous work from our group has determined that there is a wide variation in the volume of workload between trainers in Australia. Furthermore, the association between workload volume and trainer success is weak (13, 14). How these training practices affect the risk of MSI in racehorses is unknown. This study aimed to explore the relationship between race day MSI and typical intended training programs, including intended workout and rest practices of racehorses reported by Victorian Thoroughbred trainers. By assessing this relationship based on how trainers intend to train a healthy horse instead of the previous training history of the horse, the confounding effect of injury is curtailed.

## 2. Materials and methods

### 2.1. Predictor variables

A total of 66 (of 889) registered trainers in Victoria, Australia representative of all license levels (Class A, General, Restricted) and regional classifications (Metropolitan, Provincial, Country) from the 2016/2017 racing season were surveyed to determine their training programs as described in detail in a previous publication (13). Trainers were asked to provide details about rest practices, progressive (pre-trial), and race-fit training programs for healthy horses in the absence of injury or other training limiting factors for two-year-old and mature (≥three-year-old) horses, as well as for each targeted race distance category of race-fit, mature horses [sprinters (<1,300 m), middle distance (1301–2,100 m), stayers (>2,100 m)]. For several variables, trainers provided their response as a range rather than a single value. Unless otherwise stated, where a range was provided, an expected mean and standard deviation was calculated for the variable, assuming a normal distribution. For each horse within each stable, a random draw was taken from this distribution, providing an estimate of the variable for each individual horse. A trainer mean was then calculated using the vector of individual horse random draws. This approach allowed for inherent variability in the stable-level mean where the number of horses per stable was small (13). A complete list of study factors is provided in Supplementary Table 1.

#### 2.1.1. Rest variables

Trainers provided a range for how long (rest period) and how often (frequency of rest) they would rest their two-year-old and mature horses. The average rest period and frequency of rest was used for analysis. A categorical variable for rest practices was generated, accounting for both duration and frequency of rest as follows: (i) Short and less frequent – no rest, or once a year for 6 weeks or shorter per rest period; (ii) Short and more frequent – more than once a year for 6 weeks or shorter per rest period; (iii) Long and less frequent – two or fewer rest periods per year for longer than 6 weeks per rest period; (iv) Long and more frequent – more than twice a year for longer than 6 weeks per rest period. Seven trainers could not provide a definitive answer for how often they rested their two-year-old and/or mature horses and were assigned one rest period per year, the minimum (non-zero) number of rest periods per year and therefore placed in the “less frequent” rest frequency category. Three trainers did not provide a definitive answer for duration of rest periods for their two-year-old and/or mature horses and were assigned to the “long” rest period category based on the mean value of the rest period length of the other trainers (6.57 weeks for two-year-old horses and 6.33 weeks for mature horses). The classification of these missing values did not affect the outcome of univariable models.

#### 2.1.2. Progressive and race-fit training programs

Intended total race distance and galloping workloads were defined as speed of galloping in seconds per metric furlong (200 m) and summed over speed categories of 13.3–14.3 m/s, 14.4–15.4 m/s, 15.5–16.7 m/s, and ≥ 16.8 m/s for both pre-trial and race-fit horses. We defined fast work as distances (m) at speeds of 13.3 m/s and above (“total galloping”) and categorised them into four speeds (13.3–14.3 m/s “slow-speed galloping,” 14.4–15.4 m/s “medium-speed galloping,” 15.5–16.7 m/s “high-speed galloping,” ≥16.8 m/s “very high-speed galloping”). Horse workloads at the medium-speed category were not reported for 100% of trainers for two-year-old, and 94 to 97% of trainers for mature horses across all progressive and race-fit categories. These variables were therefore not considered for inclusion in multivariable analyses. Number of weeks from paddock to slow work, number of weeks to trial, number of weeks of fast workouts for progressive programs; and frequency of fast workouts and number of weeks between race starts for race-fit training programs were recorded. Cluster analyses from our previous study grouped progressive workloads into categorical programs for two-year-old horses, as well as progressive and race-fit programs for mature horses (13). This present study has adapted the cluster programs as follows: Progressive programs for two-year-old workloads – (i) Fast and light, (ii) Moderate volume, and (iii) High volume over extended time periods; Progressive programs for mature horse workloads–(i) Fast and light, (ii) Moderate volume, and (iii) High volume with slower speed gallops. Maintenance workload programs for race-fit horses were categorised as (i) Low volume, (ii) Moderate volume, and (iii) High volume. Supplementary Table 2 provides descriptions for each progressive and race-fit cluster groups. Each trainer was classified according to their primary workload level. Trainers were ranked according to the proportion of workloads in each of the described mature race-fit cluster volume groups and were assigned a “workload percentile”.

#### 2.1.3. Miscellaneous training variables

Types of surfaces used for fast and slow work exercise and whether trainers utilised alternative exercise methods (walker, treadmill, swim) were recorded. Trainers also indicated if they used a standardised or specialised training program for their horses, and if they galloped, trialled, or raced two-year-olds. For types of surfaces, trainers provided detail on their preferred percentage of fast and slow work carried out on each training surface type. For analysis, four binary (0/1) categorical variables were generated, one each for work carried out on dirt, turf, sand, and synthetic surfaces respectively; and each trainer was assigned ‘1’ in these categories if they conducted the majority of their work (highest percentage) on that surface compared to the other surfaces, and ‘0’ if it wasn’t their preferred surface. Trainers were assigned to more than one work surface variable if they had more than one preferred surface type. Training surface variables were not considered for inclusion in the multivariable models but are instead discussed descriptively due to the low numbers of injuries and trainers utilising certain surfaces.

### 2.2. Injury (outcome) data

The study period was defined as the start of the 2013/2014 racing season to the end of the 2016/2017 racing season in Victoria, Australia (1 August 2013 to 31 July 2017). Race and injury data for the study period were obtained from Racing Australia’s Single National System (SNS) and the Australian Racing Incident Database (ARID) respectively. For calculation of injury rates, denominator data were the total number of official race starts stratified by horse age (two-year-old versus ≥ three-year-old “mature horse”) per trainer involved in the survey during the study period. Entries where the horse did not start the race (scratched, race abandoned) were excluded from analysis. Numerator data were the number of race day MSI stratified by horse age (two-year-old versus ≥ three-year-old “mature horse”) per trainer during the study period. An MSI was defined as any injury classified in ARID as bone, joint, muscle, tendon, ligament, and musculoskeletal categories, as well as any additional observations of lameness. MSI was inclusive of non-catastrophic and catastrophic MSI (CMI). As there was no two-year-old CMI, all two-year-old MSI outcomes are by default non-catastrophic.

### 2.3. Data analysis

Data analyses were performed using Stata/IC version 15.1 (StataCorp LLC, College Station, TX). Two outcome variables were investigated: (i) number of MSI and (ii) number of CMI, stratified into two-year-old and mature horses. Rest and training variables for two-year-old racehorse training programs were investigated as predictors of MSI and CMI sustained by two-year-old and mature horses. Rest and training variables for mature horses were investigated as predictors for only mature horse MSI and CMI because those variables cannot influence risk of MSI occurring earlier than that training program is implemented.

Univariable negative binomial generalised linear regression models with a log-link were generated for each of the outcome variables. This models the count of MSI for each predictor, and by including the logarithm of the number of race starts as an offset, we effectively estimate the rate of MSI by trainer. Negative binomial regression was selected over Poisson regression due to overdispersion within the outcome data. That is, the variance was greater than the mean due to a high number of trainers with no MSI (*n* = 12), CMI (*n* = 53), or two-year-old MSI (*n* = 44). The appropriateness of the models was assessed using the Pearson goodness-of-fit test after running a Poisson model and confirmed by using the likelihood ratio test after fitting the negative binomial model.

The linearity of relationship between predictor and outcome variables was assessed using Box-Tidwell power transformations. Model residuals were assessed for normality using histograms. Where appropriate, continuous predictor variables were categorised. A significant outlier was detected in the CMI models and thus models were generated with and without the outlier, but as the effect was minimal, all reported analyses included that data point.

Variables with *p* ≤ 0.2 in univariable models were considered for inclusion in the multivariable models. Collinearity between variables was assessed using pairwise Pearson correlation coefficients (*r*). For variables that were collinear (*r* ≥ 0.6), the variable that minimised the Bayesian Information Criterion (BIC) in its respective univariable model was selected for inclusion in the initial multivariable models. Two-year-old progressive and race-fit variables such as progressive training categories and weeks between starts that had missing not at random (MNAR) observations, i.e., where trainers did not provide answers if they did not gallop, trial, or race two-year-olds were dealt with in multivariable analyses using a dummy variable (15). Variables were eliminated using backwards stepwise elimination and retained in the final multivariable model where *p* ≤ 0.05. Incidence rate ratios (IRR) and their 95% confidence intervals (95% CI) are reported. To determine whether combinations of programs predicted greater risk, we generated two-way interaction terms between progressive and race-fit program variables for each model. Significant interaction terms were not added to the final multivariable models due to potential overfitting but are described separately. Poisson regression was used to calculate the differences in incidence rates between the study population and that of the wider Victorian racing population.

Measures of trainer success of numbers of wins, places, and prizemoney over a trainer’s total career and previous season (1 August 2016 to 31 July 2017) were obtained from the official repository for Australian racing results, accessed in the period between 13^th^ to the 29^th^ of March 2018 as detailed in our previous study (14). Relationships between variables significant in the final multivariable models and outcome measures of trainer success were assessed univariably using negative binomial regression offset with the logarithm of the number of race starts (wins and places); linear regression (natural log of career prizemoney per start); and logistic regression (previous season prizemoney per start binarized to zero and more than zero prizemoney earned in the previous season) to determine if training practices associated with injury were also associated with trainer success. The link test was performed on the univariable models to identify any model specification errors.

## 3. Results

All trainers surveyed had at least one race start over the study period. During the study period, 19 of the 66 (28.8%) trainers did not have a two-year-old race start. At the time of survey, 58 trainers (87.9%) stated that they galloped two-year-olds, 56 trainers (84.8%) trialled two-year-olds, and 47 trainers of the 66 surveyed trainers (71.2%) raced two-year-olds. Table 1 presents a descriptive summary of the mean, standard deviation, and range of the number of race starts and MSI injuries for the study population.

**Table 1:** Descriptive statistics (mean, standard deviation, and range) of number of race starts, musculoskeletal injuries (MSI), and catastrophic musculoskeletal injuries (CMI) per trainer for *n* = 66 (100%) trainers in Victoria Australia for horses of all ages and ≥three-year-old horses; and *n* = 47 (71.2%) of trainers that recorded at least one two-year-old race start within the study period (1 August 2013 to 31 July 2017).

	Mean (sd)	Range
Number of race starts		
All ages	699.3 (1107.7)	8,7835
Two-year-old	51.8 (73.4)	1,367
≥Three-year-old	662.4 (1058.5)	8,7601
Number of MSI		
All ages	9.4 (18.8)	0,135
Two-year-old	0.7 (1.3)	0,7
≥Three-year-old	8.82 (18.1)	0,133
Number of CMI		
All ages	0.3 (0.8)	0,5
Two-year-old	0.0 (0.0)	0,0
≥Three-year-old	0.3 (0.8)	0,5

Of the 66 surveyed trainers, 12 trainers (18.2%) had no race day MSI recorded and 25 (53.2%) of the 47 trainers that raced two-year-olds had no two-year-old MSI recorded during the period of the study. A total of 53 of the 66 trainers (80.3%) did not have any CMI recorded and there were no recorded CMI for two-year-olds. There were 13.4 MSI per 1,000 race starts (95% CI 12.3, 14.5) and 0.4 CMI per 1,000 race starts (95% CI 0.3, 0.7) for the 66 trainers surveyed. There were 14.4 two-year-old MSI per 1,000 race starts (95% CI 10.0, 20.0) for the 47 trainers that recorded at least one two-year-old race start during the study period.

### 3.1. MSI univariable analyses

Univariable associations between predictor variables and injury outcomes are presented in Supplementary Table 1 for the two-year-old MSI, mature MSI, and mature CMI models. In univariable analyses, trainers who preferred to carry out most of their slow work on sand compared to other surfaces had lower mature horse MSI risk (*p* = 0.008). Trainers who preferred to carry out most of their slow work on dirt had lower two-year-old MSI (*p* < 0.001) and mature horse CMI (p < 0.001) risk, however, only one of the 47 trainers with two-year-old starters and six trainers of mature-aged starters stated that they conducted the majority of their slow work on dirt tracks. These trainers had no CMI or two-year-old MSI during the study period. Due to the low numbers of trainers that trained on specific surfaces, inferences could not be made about MSI risk based on training surfaces. Total monthly gallop distances at each speed category and targeted racehorse distance category for mature, race-fit horses were not retained in the multivariable model as they were explained by the clustered race-fit training programs.

Two-way interaction terms between progressive and race-fit grouped training practices were assessed to determine if certain combinations of progressive and mature intended training practices were associated with MSI risk, however, the number of injuries and/or trainers in each of the cluster groups were insufficient to be able to draw any inferences.

### 3.2. MSI multivariable analyses

Multivariable associations between predictor variables and two-year-old and mature MSI outcomes are presented in Table 2. Preferred rest periods of trainers in our study ranged between two to 12 weeks and two to 16 weeks for two-year-old and mature horses, respectively. Annual frequencies of rest ranged between zero to five rests for two-year-old horses and zero to four rests for mature horses. Trainers who preferred to rest their two-year-old horses more than once a year for 6 weeks or shorter per rest period had lower two-year-old MSI risk compared to trainers who preferred to rest their two-year-old horses only once a year or not at all. These trainers also had reduced MSI risk compared to trainers who preferred to rest their horses for longer lengths of time per rest period (Table 2, Two-year-old rest practices, Two-year-old MSI). The magnitude of the effect of rest practices was smaller for mature horses (Table 2). Trainers who preferred long and more frequent rest periods for mature horses (more than two rests per year for greater than 6 weeks per rest) had reduced MSI risk compared to trainers who rested their horses less frequently at greater than 6 weeks per rest (IRR 0.58; 95% CI 0.42, 0.81; *p* = 0.002) as well as trainers who preferred short and frequent rests (more than one rest per year for 6 weeks or less per rest) (Table 2, ≥Three-year-old rest practices, Mature MSI). Trainers who preferred to rest their mature horses only once a year for 6 weeks or less or not at all had lower MSI risk compared to trainers who rested their horses at a frequency of two or fewer rests per year for longer than 6 weeks per rest (IRR 0.75; 95% CI 0.56, 1.00; *p* = 0.047).

**Table 2:** Multivariable modelling of rest and training-related study factors and the risk of race day musculoskeletal injuries (MSI) for two-year-old Thoroughbred horses, and mature horses (≥ three-year-old) stratified by all injuries (catastrophic and non-catastrophic) or catastrophic (CMI) musculoskeletal injuries of 66 surveyed Victorian trainers, 2013 to 2017. Rest and training variables for mature horses were not investigated as predictors for two-year-old injuries as they cannot influence risk of injuries occurring earlier than that training program is implemented. Incidence rate ratios (IRR) and their associated 95% confidence intervals (CI) are presented; *N* = number of trainers.

	Two-year-old injuries^a^			Mature (≥Three-year-old) injuries					
	MSI^b^			MSI^c^			CMI		
Study factor	*N*	IRR (95% CI)	Value of *p*	*N*	IRR (95% CI)	Value of *p*	*N*	IRR (95% CI)	Value of *p*
*Two-year-old rest variables*									
Rest practices (Categorical)									
Short and less frequent	7	4.02 (1.70, 9.53)	0.002						
Short and more frequent	19	Ref							
Long and less frequent	12	2.36 (1.41, 3.95)	0.001						
Long and more frequent	9	3.44 (2.08, 5.70)	<0.001						
*≥Three-year-old rest variables*									
Rest practices (Categorical)									
Short and less frequent				10	0.86 (0.65, 1.13)	0.273			
Short and more frequent				28	Ref				
Long and less frequent				20	1.14 (0.93, 1.40)	0.205			
Long and more frequent				8	0.66 (0.46, 0.95)	0.026			
*Two-year-old progressive training methods*									
Training programs									
Fast and light	19	Ref							
Moderate volume	22	1.83 (1.22, 2.75)	0.003						
High volume over extended time periods	5	1.25 (0.13, 11.6)	0.847						
*≥Three-year-old progressive training methods*									
Training programs									
Fast and light				32	Ref				
Moderate volume				26	1.19 (0.95, 1.49)	0.136			
High volume with slower speed gallops				8	0.77 (0.65, 0.92)	0.004			
Time to trial (weeks)							65	55.9 (1.12, 2792.09)	0.044
Time to trial^2^ (Quadratic term)							65	0.75 (0.58, 0.96)	0.025
*Two-year-old race-fit training methods*									
Total distance galloped per month at 13.3–14.3 m/s (km)				65	1.08 (1.02, 1.14)	0.005			
Total distance galloped per month at 15.5–16.7 m/s (km)	46	0.76 (0.64, 0.90)	0.001	65	0.92 (0.86, 0.98)	0.012			
*≥Three-year-old race-fit training methods*									
Training programs									
Low volume							14	0.00 (0.00, 0.00)	<0.001
Moderate volume							46	Ref	
High volume							6	2.66 (1.17, 6.09)	0.020
*Miscellaneous training variables*									
Galloped, trialled, or raced two-year-olds									
Does not gallop, trial, or race two-year-olds				8	0.95 (0.48, 1.90)	0.895	8	0.00 (0.00, 0.00)^d^	<0.001
Does not race two-year-olds but gallops or trials them				11	0.70 (0.49, 0.99)	0.046	11	0.00 (0.00, 0.00)^d^	<0.001
Races two-year-olds				47	Ref		47	Ref^d^	

a 19 trainers did not have any two-year-old race starts within the study period and was not included in denominator data.

b No two-year-old CMI were recorded during the study period.

c Includes CMI.

d 0 CMI/8 trainers in “Does not gallop, trial, or race”, 1/11 in “Does not race but gallops or trials”, and 12/47 in “Races” two-year-olds.

Trainers who preferred to utilise fast and light progressive training programs for two-year-olds had lower two-year-old MSI risk compared to trainers who preferred moderate volume training programs (Table 2, Two-year-old progressive training programs, Two-year-old MSI). Trainers who preferred high volume with slower speed gallop progressive programs for their mature horses had lower mature horse MSI risk compared to moderate and fast and light progressive workloads, however, there were only eight trainers in the high volume with slower speed gallop category (Table 2, ≥ Three-year-old progressive training programs, Mature MSI). Progressive cluster programs and stable size were interchangeable in the mature horse multivariable model, where smaller stable sizes were associated with lower risk of mature horse MSI. The eight trainers in the high volume with slower speed gallop progressive cluster group were trainers with smaller stables, with a mean of 13.9 horses in the stable (s.d. 20.3, min 1, max 61), compared to a mean of 33 horses for moderate volume (s.d. 29.4, min 1, max 106), and 24.6 for fast and light (s.d. 33.4, min 1, max 191) progressive training program clusters.

Preferred two-year-old monthly race-fit distances at slow-speed galloping (13.3–14.3 m/s) for trainers that recorded at least one two-year-old race start during the study period ranged between zero km (not a preferred training speed; two trainers) and 8 km per month (median 3.2, IQR 1.6–4.0). Preferred two-year-old monthly distances at high-speed galloping (15.5–16.7 m/s) ranged between zero km (four trainers) and 6.4 km per month (median 2.4, IQR 1.6–3.2). Trainers who preferred higher two-year-old race-fit total monthly workloads at high-speed-galloping had lower MSI risk for both their two-year-old and mature horses (Table 2, Two-year-old race-fit training methods, Two-year-old and Mature MSI), however, preferred training volumes for two-year olds were lower than for mature horses (13). Trainers who preferred lower two-year-old race-fit monthly workloads at slow-speed galloping had lower mature horse MSI risk (Table 2, Two-year-old race-fit training methods, Mature MSI). Trainers who preferred not to train their two-year-old race-fit horses at very high-speeds (≥16.8 m/s; 37 trainers) were associated with lower two-year old MSI risk, however, this variable was removed from the final two-year-old multivariable model due to being negatively correlated with high-speed galloping (15.5–16.7 m/s).

Trainers who only preferred to gallop or trial two-year-olds had a lower risk of mature horse MSI compared to trainers who preferred to race two-year-olds (Table 2, Miscellaneous training variables, Mature MSI).

### 3.3. CMI multivariable analysis

The mean times to trial for mature horses ranged between 6 and 14 weeks. Trainers who preferred longer times to trial in mature horse programs beyond 8 weeks were associated with lower risk of CMI (Table 2, ≥ Three-year old progressive training methods, Mature CMI), with the highest risk of CMI associated with trainers whose preferred times to trial were between 6 and 8 weeks. Trainers who preferred low or moderate volume race-fit mature horse programs had lower risk of CMI compared to trainers who preferred a high volume training program, however, only six of the surveyed trainers (9.1%) fell into the high volume race-fit category (Table 2, ≥ Three-year-old race-fit training methods, Mature CMI). CMI risk in mature horses was lower for trainers who preferred not to race two-year-olds (one CMI from 19 trainers that preferred not to race two-year olds compared to 12 CMI from 47 trainers that raced two-year-olds) (Table 2, Miscellaneous training variables, mature CMI).

### 3.4. Performance univariable analyses

In general, preferred training practices that were associated with lower MSI and/or CMI risk in their respective models were either not significantly associated, or associated with better trainer performance. One exception was that trainers who preferred not to race two-year-olds were associated with lower performance univariably but also a lower risk of MSI and CMI in the mature horse models (Supplementary Table 3).

## 4. Discussion

In this study we examined how trainers’ attitudes to training practices for Thoroughbred racehorses were associated with MSI identified on race day. We found differing effects of two-year-old and mature horse training practices. Lower two-year-old race day MSI risk was associated with trainers who preferred shorter times to trial, shorter time in fast work and lower cumulative gallop distances pre-trial, as well as short, frequent rests. Conversely, lower MSI risk in the mature horse was associated with trainers who preferred a high-volume pre-trial training strategy comprising a high volume of slower gallop training and longer times to trial. How a trainer prefers to train their race-fit two-year-old horses was associated with their horse’s MSI risk later in their career, with more 15.5–16.7 m/s but not ≥16.8 m/s galloping work associated with lower MSI risk. Also, there was a decreased risk of MSI later in a horse’s career for trainers who preferred not to race two-year-olds. Trainers who preferred longer times to trial and low volume race-fit training programs for mature horses had a lower risk of CMI while a high-volume race-fit training program was associated with increased CMI risk.

Although rest periods are critical for enhancing bone repair, as demonstrated by microstructural studies of subchondral bone (16), the risk of injury is higher when horses return to training following a rest period, which may explain the varied findings in available literature (4). A Californian study has previously demonstrated that a hazard period of 10 days following >60 days rest was found to be associated with an increased risk of humeral fracture, which is in line with our result that trainers who preferred shorter rests (6 weeks or less) had a lower occurrence of MSI in their younger horses (17). In contrast to younger horses, our study has found that for mature horse training programs, trainers who preferred longer rests were associated with lower MSI risk when horses were rested frequently. Mathematical models have demonstrated that the state of the subchondral bone at the time the horse commences its rest period is important as horses that have higher initial bone volume fraction, i.e., horses that have undergone more intensive training, obtain less benefit from the same period of rest in terms of bone remodelling compared to horses with lower bone volume fraction (18). Therefore, we hypothesise that shorter rests may optimise the benefits of rest periods in two-year-old horses whose skeletons are undergoing adaptation, however, in older horses, longer periods of rests are more tolerated and perhaps necessary as their bones, while better adapted, have accumulated more potential damage thereby needing repair (19).

In our study, trainers who preferred more frequent rests overall were associated with lower two-year-old and mature horse MSI risk. The benefit of higher frequencies of rests for younger horses was in accordance with results from a prospective case–control study in Queensland, Australia which found that a longer training period without rest was associated with an increased MSI risk in two-year-old horses (20). However, the Queensland study did not observe the same finding for older horses. A greater time since the last rest period was also found to be associated with catastrophic metacarpal condylar and proximal sesamoid bone fracture (4). Our findings highlight the importance of implementing tailored training practices to suit the needs of the individual horse, where an adequate duration of rest is likely to be dependent on the duration and intensity of the previous training period, bone turnover rates, and the volume of damaged bone present (16, 18, 19, 21). Duration and frequency of rests were not significant factors for CMI, however power to detect differences in this model was low due to the low prevalence of CMI.

Our study findings are consistent with the concept that gallop work is beneficial for horses of all ages, however, the training needs of horses of varying ages and fitness levels appears to differ. Previous research has documented the importance of gallop work for horses in training. For example, a study of risk factors for fatal distal limb fracture in racing Thoroughbreds in the UK found that horses that did no gallop work during training were at an increased risk of fracture (22). Similarly, a study conducted in the UK monitoring a cohort of 1,178 Thoroughbreds for up to 2 years demonstrated that in previously untrained horses, gradual accumulation of high-speed exercise had a protective effect on fracture risk (7).

The differences in progressive training practices associated with MSI risk in two-year-old and mature horses highlights that it is important to adapt pre-trial training programs as a horse progresses along its career. In younger horses, a pre-trial training program that incorporates sufficient high speed work to stimulate adaptation while avoiding skeletal damage associated with excessive galloping volumes is crucial (i.e., the “fast and light” training program) as the skeletons of younger horses are still developing, demonstrated by an increase in bone density in the MCIII condyles in two-year-old trained versus untrained horses (23). In contrast, when an older horse undergoes a period of rest, i.e., bone de-adaptation (19), a subsequent pre-trial training method that takes into account a slower bone adaptation response, incorporating high volumes of both slow and fast gallops over longer times to trial (i.e., “High volume with slower speed gallops”) may be more appropriate. However, only 8 of the 66 trainers surveyed (12%) preferred this pre-trial training regime for their mature horses, and as they were also trainers with smaller stables, it was unclear if the lower mature horse MSI risk was attributable to the training program or a particular characteristic of trainers from smaller stables. Our previous study had found that smaller stable sizes were associated with lower rates of career wins and places as well as career and last season prizemoney per start therefore it might be the case that trainers from smaller stables are more risk averse in their overall training methodology, contributing to both lower MSI incidence and performance (14).

On the whole, trainers prescribed more conservative race-fit training volumes for their two-year-old horses compared to mature horses (13). Our finding that trainers who preferred higher volumes of high speed work for their two-year-old race-fit horses experienced lower MSI risk in their two-year-old year and later in their careers is in line with existing literature showing a lack of negative effects and some positive effects of early conditioning exercise in Thoroughbreds (4, 24, 25). The lower MSI risk associated with trainers who prefer not to train their two-year-old horses at very high speeds (≥ 16.8 m/s), while not retained in the final two-year-old multivariable model, may perhaps be an indication that while some high speed work may be necessary to adapt the bones of younger horses to racing, this has to be carried out in moderation. However, our finding that trainers who preferred not to race two-year-olds were associated with a lower risk of MSI and CMI along with lower performance univariably in the mature horse models could indicate that these trainers are more careful in other aspects of their horse management, but we are unable to confirm this based on available data. In mature race-fit horses, our finding that trainers who preferred high volumes of fast work were associated with greater CMI risk is consistent with the need to limit fast work to avoid the accumulation of microdamage that may exceed the capacity of mature bone (5). This result is in line with previous research in California showing that high average daily rates of exercise distance accumulation within a 2-month period was associated with greater risk of CMI (26). Additionally, increased training gallops in the most recent season was observed to be associated with an increased risk of higher grade palmar osteochondral disease in a study of 164 Thoroughbred horses examined at post-mortem, supporting the idea that prolonged, cyclical loading at high stress levels is likely to result in failure of bone (27).

Apart from trainers’ preference on whether to race two-year-olds discussed above, this study has found that preferred training practices that minimize the risk of MSI do not negatively affect the performance of the racehorse or their earnings. This demonstrates that there is room for the modification of workload intensity to minimize the likelihood of MSI without the risk of compromising performance, which is in agreement with results from our previous study (14).

As training program data was obtained via interviews, this study is subject to misclassification and recall biases. However, in-person interviewing enabled greater detail to be collected and minimised nonresponse errors (14). This study is also subject to potential selection bias as the interviews were conducted on a voluntary basis thus the findings of the study may not be generalisable to other populations of trainers, however, the incidence of 0.4 CMI per 1,000 race starts in this group of surveyed trainers was similar to a pooled CMI incidence for Australia and New Zealand in previous decades (4), an indication that the findings of this study is a fair representation of the overall population. We have no knowledge of the surveyed trainers’ attitudes to medication, their risk thresholds, or level of veterinary input; all of which are potential confounders in this study. Due to the large volume of data collected from participants across varied aspects of intended training programs and conditions, there were some multi-collinearity of predictor variables. Where choosing between collinear variables based on clinical relevance was not possible, the variables were chosen based on consistent statistical methods. Due to a limited statistical power, assessing interactions between variables was challenging and no inferences could be made on the possible interplay between variables affecting MSI risk. Additionally, caution needs to be taken when interpreting the results of the multivariable CMI model due to the low proportion of trainers surveyed recording a CMI during the study period (19.7%). As occurrences of training MSI are not routinely recorded, data for this study was limited to race day MSI, hence, any MSI that may have occurred during training including prior to a horse’s first career race were not considered.

Understanding the relationship between training practices and MSI will inform the development of training methods that will improve the welfare and safety of horses in the racing industry. We have demonstrated the importance of tailoring a horse’s training practices as it progresses along its career to minimize musculoskeletal injury risk. Our study findings indicate that younger horses may benefit from shorter and more frequent rests along with a conservative approach towards gallop work pre-trial. Mature horses may benefit from longer times to trial and higher volumes of galloping work pre-trial which include higher volumes of slower gallop speeds. In both young and mature race-fit horses, high-speed gallop work is necessary but should be moderated to avoid the excessive accumulation of microdamage that might occur. Encouragingly, preferred training practices that were associated with the minimization of MSI risk did not compromise trainer performance. Further work is needed to investigate how combinations of certain rest, progressive, and race-fit training practices may interact in contributing to MSI risk.

## Data availability statement

The datasets presented in this article are not readily available because of privacy reasons but may be available upon request from the corresponding author. Requests to access the datasets should be directed to AW, adelenew@unimelb.edu.au.

## Ethics statement

The studies involving humans were approved by the Faculty of Veterinary and Agricultural Sciences Human Ethics Committee at The University of Melbourne (reference 1647911.1). The studies were conducted in accordance with the local legislation and institutional requirements. The participants provided their written informed consent to participate in this study.

## Author contributions

AW: Data curation, Formal analysis, Investigation, Methodology, Writing – original draft, Writing – review & editing. AM-W: Conceptualization, Data curation, Formal analysis, Investigation, Methodology, Writing – review & editing. PH: Formal analysis, Investigation, Methodology, Writing – review & editing, Supervision.RW: Conceptualization, Methodology, Writing – review & editing, Supervision.
