# Supplementary material for: The association between Thoroughbred racehorse training practices and musculoskeletal injuries in Victoria, Australia
Source: Front Vet Sci. 2023 Oct 24;10:1260554. doi: 10.3389/fvets.2023.1260554 (PMC10628463; doi:10.3389/fvets.2023.1260554)
Supplement: Supplementary file 1 [file Table_1.DOCX]

Supplementary Table 1. Univariable association between rest and training-related study factors and the risk of race day musculoskeletal injuries (MSI), stratified by age, and all injuries (catastrophic and non-catastrophic) or catastrophic (CMI) musculoskeletal injuries of Victorian trainers, 2013 to 2017. Rest and training variables for mature horses were not investigated as predictors for two-year-old injuries as they cannot influence risk of injuries occurring earlier than that training program is implemented (green shaded areas). Incidence rate ratios (IRR) and their associated 95% confidence intervals (CI) are presented.

|  | **Two-year-old injuries^a^** | | | | **Mature (≥ Three-year-old) injuries** | | | | | |
| --- | --- | --- | --- | --- | --- | --- | --- | --- | --- | --- |
|  | **MSI^b^** | | | | **MSI** | | | **CMI** | | |
| **Study factor** | **n** | | **IRR (95% CI)** | ***p*-value** | **n** | **IRR (95% CI)** | ***p*-value** | **n** | **IRR (95% CI)** | ***p*-value** |
| **Two-year-old rest variables** |  | |  |  |  |  |  |  |  |  |
| Average frequency of rest per year | 47 | | 1.05 (0.75, 1.48) | 0.767 | 66 | 1.04 (0.89, 1.21) | 0.605 | 66 | 1.08 (0.57, 2.04) | 0.825 |
| Average frequency of rest per year (Categorical) |  | |  |  |  |  |  |  |  |  |
| ≤ 1 rest | 10 | | Ref |  | 18 | Ref |  | 18 | Ref |  |
| >1, ≤ 2 rests | 19 | | 0.58 (0.22, 1.54) | 0.277 | 25 | 1.30 (0.98, 1.73) | 0.070 | 25 | 1.70 (0.41, 7.09) | 0.465 |
| > 2 rests | 18 | | 0.59 (0.22, 1.60) | 0.303 | 23 | 1.11 (0.80, 1.54) | 0.546 | 23 | 1.14 (0.25, 5.24) | 0.862 |
| Average rest period (weeks) | 47 | | 1.27 (1.07, 1.53) | 0.008 | 66 | 0.98 (0.94, 1.03) | 0.384 | 66 | 0.93 (0.70, 1.22) | 0.588 |
| Rest practices (Categorical) |  | |  |  |  |  |  |  |  |  |
| Short and less frequent | 7 | | Ref |  | 8 | Ref |  | 8 | Ref |  |
| Short and more frequent | 19 | | 0.28 (0.09, 0.91) | 0.034 | 24 | 1.25 (0.83, 1.88) | 0.288 | 24 | 2.15 (0.36, 12.7) | 0.398 |
| Long and less frequent | 12 | | 0.74 (0.23, 2.36) | 0.613 | 22 | 1.30 (0.86, 1.97) | 0.206 | 22 | 1.29 (0.16, 10.0) | 0.810 |
| Long and more frequent | 9 | | 0.85 (0.29, 2.53) | 0.769 | 12 | 1.07 (0.64, 1.78) | 0.790 | 12 | 1.13 (0.14, 9.51) | 0.908 |
| **≥ Three-year-old rest variables** |  | |  |  |  |  |  |  |  |  |
| Average frequency of rest per year |  | |  |  | 66 | 0.87 (0.73, 1.03) | 0.113 | 66 | 0.90 (0.29, 2.81) | 0.862 |
| Average frequency of rest per year (Categorical) |  | |  |  |  |  |  |  |  |  |
| ≤ 1 rest |  | |  |  | 19 | Ref |  | 19 | Ref |  |
| >1, ≤ 2 rests |  | |  |  | 27 | 1.16 (0.88, 1.53) | 0.303 | 27 | 0.86 (0.24, 3.12) | 0.815 |
| > 2 rests |  | |  |  | 20 | 0.79 (0.56, 1.10) | 0.167 | 20 | 0.54 (0.11, 2.63) | 0.446 |
| Average rest period (weeks) |  | |  |  | 66 | 1.04 (0.99, 1.09) | 0.105 | 66 | 0.76 (0.54, 1.07) | 0.113 |
| Rest practices (Categorical) |  | |  |  |  |  |  |  |  |  |
| Short and less frequent |  | |  |  | 10 | Ref |  | 10 | Ref |  |
| Short and more frequent |  | |  |  | 28 | 0.95 (0.68, 1.33) | 0.753 | 28 | 0.89 (0.30, 2.67) | 0.838 |
| Long and less frequent |  | |  |  | 20 | 1.24 (0.88, 1.74) | 0.221 | 20 | 0.17 (0.02, 1.62) | 0.122 |
| Long and more frequent |  | |  |  | 8 | 0.56 (0.32, 0.97) | 0.038 | 8 | 0.81 (0.12, 5.64) | 0.831 |
| **Two-year-old progressive training methods** |  | |  |  |  |  |  |  |  |  |
| Training programs |  | |  |  |  |  |  |  |  |  |
| Fast and light | 19 | | 3.53 (0.45, 28.05) | 0.232 | 23 | Ref |  | 23 | Ref |  |
| Moderate volume | 22 | | 4.22 (0.55, 32.25) | 0.166 | 26 | 1.05 (0.81, 1.37) | 0.698 | 26 | 0.82 (0.28, 2.38) | 0.720 |
| High volume over extended time periods | 5 | | Ref |  | 7 | 1.21 (0.95, 1.54) | 0.118 | 7 | 0.41 (0.07, 2.41) | 0.324 |
| Time to trial (weeks) | 44 | | 1.03 (0.83, 1.27) | 0.808 | 55 | 1.04 (0.99, 1.10) | 0.136 | 55 | 0.86 (0.68, 1.08) | 0.201 |
| Time from paddock to slow work (weeks) | 46 | | 1.34 (1.01, 1.77) | 0.044 | 60 | 1.09 (1.02, 1.18) | 0.014 | 60 | 0.87 (0.63, 1.21) | 0.419 |
| Time from paddock to slow work (categorical) |  | |  |  |  |  |  |  |  |  |
| ≤ 5 weeks | 33 | | Ref |  | 44 | Ref |  | 44 | Ref |  |
| > 5 weeks | 13 | | 1.64 (0.90, 2.98) | 0.107 | 16 | 1.43 (1.13, 1.79) | 0.003 | 16 | 0.60 (0.19, 1.87) | 0.380 |
| Time in fast work pre trial (weeks) | 46 | | 0.90 (0.70, 1.16) | 0.431 | 56 | 1.00 (0.95, 1.05) | 0.970 | 56 | 0.92 (0.74, 1.16) | 0.499 |
| Total gallop distance pre trial at (> 13.3 m/s) (km) | 47 | | 0.91 (0.81, 1.04) | 0.156 | 59 | 1.02 (0.99, 1.04) | 0.181 | 59 | 1.01 (0.90, 1.13) | 0.856 |
| Total gallop distance pre trial (> 13.3 m/s) (categorical) |  | |  |  |  |  |  |  |  |  |
| ≤ 10 km | 41 | | Ref |  | 51 | Ref |  | 51 | Ref |  |
| > 10 km | 6 | | 0.47 (0.11, 2.08) | 0.321 | 8 | 1.08 (0.84, 1.39) | 0.565 | 8 | 0.93 (0.24, 3.57) | 0.912 |
| Total distance at 13.3-14.3 m/s pre trial (km) | 47 | | 0.87 (0.75, 1.02) | 0.082 | 59 | 1.03 (1.00, 1.07) | 0.061 | 59 | 1.01 (0.87, 1.18) | 0.867 |
| Total distance at 14.4-15.4 m/s pre trial (km) | N/A | |  |  | N/A |  |  | N/A |  |  |
| Total distance at 15.5-16.7 m/s pre trial (km) | 47 | | 0.82 (0.60, 1.10) | 0.188 | 59 | 0.98 (0.91, 1.05) | 0.583 | 59 | 1.03 (0.78, 1.34) | 0.848 |
| Total distance at > 16.8 m/s pre trial (km) | 47 | | 1.32 (0.99, 1.77) | 0.058 | 59 | 1.07 (0.94, 1.23) | 0.284 | 59 | 0.95 (0.46, 1.96) | 0.897 |
| Average weekly gallop distance pre trial (>13.3 m/s) km | 46 | | 0.83 (0.34, 1.99) | 0.673 | 56 | 1.03 (0.84, 1.27) | 0.774 | 56 | 1.21 (0.49, 2.98) | 0.679 |
| Average weekly distance at 13.3-14.3 m/s pre trial (km) | 46 | | 0.79 (0.28, 2.23) | 0.657 | 56 | 1.16 (0.91, 1.49) | 0.235 | 56 | 1.35 (0.46, 3.93) | 0.586 |
| Average weekly distance at 14.4-15.4 m/s pre trial (km) | N/A | |  |  | N/A |  |  | N/A |  |  |
| Average weekly distance at 15.5-16.7 m/s pre trial (km) | 46 | | 0.28 (0.05, 1.59) | 0.152 | 56 | 0.65 (0.40, 1.06) | 0.084 | 56 | 1.17 (0.26, 5.28) | 0.834 |
| Average weekly distance at > 16.8 m/s pre trial (km) | 46 | | 3.96 (1.61, 9.74) | 0.003 | 56 | 1.28 (0.79, 2.07) | 0.312 | 56 | 0.37 (0.01, 11.48) | 0.567 |
| Average weekly distance at > 16.8 m/s pre trial (km) (Categorical) |  | |  |  |  |  |  |  |  |  |
| 0 km | 37 | | Ref |  | 45 | Ref |  | 45 | Ref |  |
| > 0 km | 9 | | 1.34 (0.70, 2.58) | 0.372 | 11 | 1.06 (0.82, 1.37) | 0.654 | 11 | 1.85 (0.49, 7.03) | 0.363 |
| **≥ Three-year-old progressive training methods** |  | |  |  |  |  |  |  |  |  |
| Training programs |  | |  |  |  |  |  |  |  |  |
| Fast and light |  | |  |  | 32 | 0.80 (0.63, 1.00) | 0.051 | 32 | 1.39 (0.17, 11.22) | 0.754 |
| Moderate workload |  | |  |  | 26 | Ref |  | 26 | Ref |  |
| High volume with slower speed gallops |  | |  |  | 8 | 0.70 (0.43, 1.12) | 0.134 | 8 | 1.59 (0.07, 36.36) | 0.770 |
| Time to trial (weeks) |  | |  |  | 65 | 0.98 (0.92, 1.05) | 0.580 | 65 | 0.57 (0.46, 0.71) | 0.000 |
| Time from paddock to slow work (weeks) |  | |  |  | 65 | 0.95 (0.83, 1.09) | 0.472 | 65 | 1.32 (0.94, 1.86) | 0.105 |
| Time in fast work pre trial (weeks) |  | |  |  | 65 | 1.00 (0.94, 1.07) | 0.952 | 65 | 0.67 (0.56, 0.81) | 0.000 |
| Total gallop distance pre trial at (> 13.3 m/s)(km) |  | |  |  | 65 | 1.00 (0.98, 1.03) | 0.714 | 65 | 0.98 (0.84, 1.14) | 0.785 |
| Total gallop distance pre trial (> 13.3 m/s)(categorical) |  | |  |  |  |  |  |  |  |  |
| ≤ 10 km |  | |  |  | 43 | Ref |  | 43 | Ref |  |
| > 10 km |  | |  |  | 22 | 1.01 (0.81, 1.27) | 0.909 | 22 | 0.78 (0.25, 2.47) | 0.672 |
| Total distance at 13.3-14.3 m/s pre trial (km) |  | |  |  | 65 | 0.99 (0.96, 1.02) | 0.606 | 65 | 0.97 (0.77, 1.23) | 0.812 |
| Total distance at 14.4-15.4 m/s pre trial (km) |  | |  |  | 65 | 1.07 (0.96, 1.19) | 0.225 | 65 | 1.26 (0.94, 1.69) | 0.123 |
| Total distance at 14.4-15.4 m/s pre trial (categorical) |  | |  |  |  |  |  |  |  |  |
| 0 km |  | |  |  | 61 | Ref |  | 61 | Ref |  |
| ≥ 0 km |  | |  |  | 4 | 1.04 (0.73, 1.47) | 0.836 | 4 | 1.44 (0.76, 2.72) | 0.266 |
| Total distance at 15.5-16.7 m/s pre trial (km) |  | |  |  | 65 | 1.01 (0.95, 1.08) | 0.672 | 65 | 1.08 (0.74, 1.58) | 0.695 |
| Total distance at > 16.8 m/s pre trial (km) |  | |  |  | 65 | 1.06 (0.99, 1.15) | 0.116 | 65 | 0.84 (0.55, 1.27) | 0.400 |
| Average weekly gallop distance pre trial (>13.3 m/s) (km) |  | |  |  | 65 | 1.02 (0.86, 1.20) | 0.838 | 65 | 2.00 (1.42, 2.83) | 0.000 |
| Average weekly gallop distance pre trial (>13.3 m/s) (categorical) |  | |  |  |  |  |  |  |  |  |
| ≤ 2 km |  | |  |  | 43 | Ref |  | 43 | Ref |  |
| > 2 km |  | |  |  | 22 | 1.04 (0.82, 1.34) | 0.730 | 22 | 1.45 (0.51, 4.07) | 0.485 |
| Average weekly distance at 13.3-14.3 m/s pre trial (km) |  | |  |  | 65 | 0.91 (0.76, 1.10) | 0.320 | 65 | 1.65 (1.04, 2.61) | 0.033 |
| Average weekly distance at 13.3-14.3 m/s pre trial (categorical) |  | |  |  |  |  |  |  |  |  |
| ≤ 2 km |  | |  |  | 60 | Ref |  | 60 | Ref |  |
| > 2 km |  | |  |  | 5 | 0.45 (0.33, 0.62) | 0.000 | 5 | 3.11 (1.19, 8.11) | 0.020 |
| Average weekly distance at 14.4-15.4 m/s pre trial (km) |  | |  |  | 65 | 1.53 (0.76, 3.05) | 0.231 | 65 | 4.45 (0.66, 29.9) | 0.125 |
| Average weekly distance at 14.4-15.4 m/s pre trial (categorical) |  | |  |  |  |  |  |  |  |  |
| 0 km |  | |  |  | 61 | Ref |  | 61 | Ref |  |
| > 0 km |  | |  |  | 4 | 1.04 (0.73, 1.47) | 0.836 | 4 | 1.44 (0.76, 2.72) | 0.266 |
| Average weekly distance at 15.5-16.7 m/s pre trial (km) |  | |  |  | 65 | 1.10 (0.81, 1.50) | 0.530 | 65 | 2.52 (1.16, 5.46) | 0.019 |
| Average weekly distance at > 16.8 m/s pre trial (km) |  | |  |  | 65 | 1.63 (1.01, 2.64) | 0.045 | 65 | 0.51 (0.04, 6.49) | 0.605 |
| Average weekly distance at > 16.8 m/s pre trial (Categorical) |  | |  |  |  |  |  |  |  |  |
| 0 km |  | |  |  | 58 | Ref |  | 58 | Ref |  |
| > 0 km |  | |  |  | 7 | 1.48 (1.28, 1.71) | 0.000 | 7 | 0.77 (0.22, 2.67) | 0.676 |
| **Two-year-old race-fit training methods** |  | |  |  |  |  |  |  |  |  |
| Total distance galloped per month (km) | 46 | | 1.01 (0.86, 1.19) | 0.86 | 57 | 1.02 (0.99, 1.06) | 0.161 | 57 | 1.02 (0.84, 1.24) | 0.824 |
| Total distance galloped per month at 13.3-14.3 m/s (km) | 46 | 1.04 (0.86, 1.26) | | 0.660 | 57 | 1.08 (1.02, 1.13) | 0.004 | 57 | 1.09 (0.75, 1.59) | 0.657 |
| Total distance galloped per month at 14.4 m/s – 15.4 m/s (km) | N/A |  | |  | N/A |  |  | N/A |  |  |
| Total distance galloped per month at 15.5 – 16.7 m/s (km) | 46 | | 0.81 (0.65, 1.00) | 0.049 | 57 | 0.93 (0.87, 1.00) | 0.043 | 57 | 1.01 (0.80, 1.27) | 0.943 |
| Total distance galloped per month at >16.8 m/s (km) | 46 | | 1.13 (1.06, 1.20) | 0.000 | 57 | 1.02 (0.99, 1.06) | 0.204 | 57 | 0.90 (0.65, 1.24) | 0.530 |
| Total distance galloped per month at >16.8 m/s (km) (Categorical) |  | |  |  |  |  |  |  |  |  |
| 0 km | 37 | | Ref |  | 46 | Ref |  | 46 | Ref |  |
| > 0 km | 9 | | 1.32 (0.69, 2.53) | 0.400 | 11 | 1.08 (0.83, 1.40) | 0.581 | 11 | 1.84 (0.48, 7.13) | 0.375 |
| Average frequency of fastwork per week | 46 | | 1.09 (0.55, 2.13) | 0.810 | 57 | 0.97 (0.84, 1.13) | 0.713 | 57 | 1.13 (0.45, 2.86) | 0.799 |
| Number of weeks between racestarts | 47 | | 1.78 (1.05, 3.02) | 0.033 | 65 | 1.33 (1.09, 1.64) | 0.006 | 65 | 1.11 (0.14, 8.63) | 0.921 |
| **≥ Three-year-old race-fit training methods** |  | |  |  |  |  |  |  |  |  |
| Training programs |  | |  |  |  |  |  |  |  |  |
| Low volume |  | |  |  | 14 | 0.90 (0.67, 1.23) | 0.518 | 14 | 0.00 (0.00, 0.00) | 0.000 |
| Moderate volume |  | |  |  | 46 | Ref |  | 46 | Ref |  |
| High volume |  | |  |  | 6 | 0.99 (0.68, 1.44) | 0.946 | 6 | 2.16 (1.02, 4.55) | 0.043 |
| Workload percentile (continuous) |  | |  |  | 66 | 1.00 (1.00, 1.00) | 0.730 | 66 | 1.02 (1.00, 1.03) | 0.011 |
| Workload percentile (categorical) |  | |  |  |  |  |  |  |  |  |
| < 25% |  | |  |  | 12 | Ref |  | 12 | 0.00 (0.00, 0.00) | 0.000 |
| ≥ 25%, ≤ 75% |  | |  |  | 40 | 1.45 (1.09, 1.93) | 0.012 | 40 | 0.79 (0.28, 2.17) | 0.641 |
| > 75% |  | |  |  | 14 | 1.15 (0.81, 1.65) | 0.436 | 14 | Ref |  |
| Total distance galloped per month (km) |  | |  |  | 62 | 1.00 (0.98, 1.03) | 0.753 | 62 | 1.14 (1.05, 1.24) | 0.001 |
| Total distance galloped per month (Categorical) |  | |  |  |  |  |  |  |  |  |
| ≤ 8 km |  | |  |  | 31 | Ref |  | 31 | Ref |  |
| > 8 km, ≤ 14 km |  | |  |  | 23 | 1.22 (0.94, 1.56) | 0.131 | 23 | 2.79 (0.86, 8.98) | 0.086 |
| > 14 km |  | |  |  | 8 | 1.09 (0.76, 1.56) | 0.633 | 8 | 4.43 (1.12, 17.48) | 0.033 |
| Total monthly distance at 13.3-14.3 m/s (km) |  | |  |  | 62 | 1.00 (0.96, 1.03) | 0.799 | 62 | 1.10 (0.98, 1.23) | 0.114 |
| Total monthly distance at 13.3-14.3 m/s (Categorical) |  | |  |  |  |  |  |  |  |  |
| ≤ 5 km |  | |  |  | 39 | 0.88 (0.69, 1.12) | 0.300 | 39 | 3.78 (0.46, 30.83) | 0.214 |
| > 5 km, ≤ 10 km |  | |  |  | 18 | Ref |  | 18 | Ref |  |
| > 10 km |  | |  |  | 5 | 0.79 (0.47, 1.33) | 0.383 | 5 | 11.92 (1.51, 94.10) | 0.019 |
| Total monthly distance at 14.4-15.4 m/s (km) |  | |  |  | 62 | 1.04 (1.01, 1.07) | 0.014 | 62 | 1.12 (1.00, 1.24) | 0.044 |
| Total monthly distance at 14.4-15.4 m/s (Categorical) |  | |  |  |  |  |  |  |  |  |
| 0 km |  | |  |  | 59 | Ref |  | 59 | Ref |  |
| > 0 km |  | |  |  | 3 | 1.09 (0.80, 1.50) | 0.580 | 3 | 1.59 (0.94, 2.70) | 0.087 |
| Total monthly distance at 15.5-16.7 m/s (km) |  | |  |  | 62 | 1.00 (0.94, 1.06) | 0.974 | 62 | 1.12 (0.93, 1.36) | 0.244 |
| Total monthly distance at 15.5-16.7 m/s (Categorical) |  | |  |  |  |  |  |  |  |  |
| ≤ 3 km |  | |  |  | 20 | Ref |  | 20 | Ref |  |
| > 3 km, ≤ 6 km |  | |  |  | 36 | 1.04 (0.81, 1.35) | 0.741 | 36 | 2.48 (0.52, 11.75) | 0.254 |
| > 6 km |  | |  |  | 6 | 1.26 (0.91, 1.74) | 0.169 | 6 | 3.11 (0.77, 12.67) | 0.113 |
| Total monthly distance at > 16.8 m/s (km) |  | |  |  | 62 | 1.23 (1.10, 1.37) | 0.000 | 62 | 1.15 (0.74, 1.76) | 0.536 |
| Total monthly distance at > 16.8 m/s (Categorical) |  | |  |  |  |  |  |  |  |  |
| 0 km |  | |  |  | 56 | Ref |  | 56 | Ref |  |
| > 0 km |  | |  |  | 6 | 1.57 (1.39, 1.77) | 0.000 | 6 | 1.26 (0.50, 3.15) | 0.624 |
| Total distance galloped per month - Sprinters (<1300m) (km) |  | |  |  | 61 | 1.01 (0.99, 1.04) | 0.375 | 61 | 1.12 (1.00, 1.25) | 0.055 |
| Total monthly distance at 13.3-14.3 m/s - Sprinters (<1300m) (km) |  | |  |  | 61 | 1.02 (0.99, 1.06) | 0.244 | 61 | 1.11 (0.98, 1.26) | 0.097 |
| Total monthly distance at 14.4-15.4 m/s - Sprinters (<1300m) (km) |  | |  |  | 61 | 0.91 (0.86, 0.95) | 0.000 | 61 | 1.16 (1.09, 1.24) | 0.000 |
| Total monthly distance at 15.5-16.7 m/s - Sprinters (<1300m) (km) |  | |  |  | 61 | 0.98 (0.93, 1.03) | 0.414 | 61 | 1.15 (0.95, 1.39) | 0.142 |
| Total monthly distance at > 16.8 m/s - Sprinters (<1300m) (km) |  | |  |  | 61 | 1.06 (1.00, 1.12) | 0.071 | 61 | 0.80 (0.57, 1.14) | 0.222 |
| Total monthly distance at > 16.8 m/s - Sprinters (<1300m) (Categorical) |  | |  |  |  |  |  |  |  |  |
| 0 km |  | |  |  | 50 | Ref |  | 50 | Ref |  |
| > 0 km |  | |  |  | 11 | 1.45 (1.23, 1.70) | 0.000 | 11 | 1.13 (0.34, 3.78) | 0.840 |
| Total distance galloped per month - middle distance horses (1301-2100m) (km) |  | |  |  | 64 | 1.02 (1.00, 1.05) | 0.066 | 64 | 1.11 (1.03, 1.20) | 0.01 |
| Total monthly distance at 13.3-14.3 m/s - middle distance horses (1301-2100m) (km) |  | |  |  | 64 | 1.02 (0.98, 1.05) | 0.309 | 64 | 1.11 (1.02, 1.22) | 0.022 |
| Total monthly distance at 14.4-15.4 m/s - middle distance horses (1301-2100m) (km) |  | |  |  | 64 | 1.07 (0.95, 1.19) | 0.268 | 64 | 0.00 (0.00, 0.01) | 0.000 |
| Total monthly distance at 15.5-16.7 m/s - middle distance horses (1301-2100m) (km) |  | |  |  | 64 | 0.99 (0.94, 1.05) | 0.733 | 64 | 1.22 (1.00, 1.48) | 0.053 |
| Total monthly distance at > 16.8 m/s - middle distance horses (1301-2100m) (km) |  | |  |  | 64 | 1.05 (0.99, 1.10) | 0.084 | 64 | 0.73 (0.49, 1.09) | 0.127 |
| Total monthly distance at > 16.8 m/s - middle distance horses (1301-2100m) (Categorical) |  | |  |  |  |  |  |  |  |  |
| 0 km |  | |  |  | 52 | Ref |  | 52 | Ref |  |
| > 0 km |  | |  |  | 12 | 1.31 (1.02, 1.68) | 0.035 | 12 | 0.75 (0.24, 2.39) | 0.633 |
| Total distance galloped per month - stayers (>2100m) (km) |  | |  |  | 64 | 1.02 (1.00, 1.04) | 0.027 | 64 | 1.09 (1.03, 1.16) | 0.006 |
| Total monthly distance at 13.3-14.3 m/s - stayers (>2100m) (km) |  | |  |  | 64 | 1.01 (0.98, 1.04) | 0.512 | 64 | 1.15 (1.04, 1.27) | 0.005 |
| Total monthly distance at 14.4-15.4 m/s - stayers (>2100m) (km) |  | |  |  | 64 | 1.02 (0.84, 1.24) | 0.821 | 64 | 0.00 (0.00, 0.00) | 0.000 |
| Total monthly distance at 14.4-15.4 m/s - stayers (>2100m) (Categorical) |  | |  |  |  |  |  |  |  |  |
| 0 km |  | |  |  | 61 | Ref |  | 61 | Ref |  |
| > 0 km |  | |  |  | 3 | 0.97 (0.54, 1.74) | 0.909 | 3 | 0.00 (0.00, 0.00) | 0.000 |
| Total monthly distance at 15.5-16.7 m/s - stayers (>2100m) (km) |  | |  |  | 64 | 1.01 (0.98, 1.05) | 0.414 | 64 | 1.06 (0.97, 1.15) | 0.181 |
| Total monthly distance at 15.5-16.7 m/s - stayers (>2100m) (Categorical) |  | |  |  |  |  |  |  |  |  |
| < 6 km |  | |  |  | 53 | Ref |  | 53 | Ref |  |
| ≥ 6 km |  | |  |  | 11 | 0.96 (0.68, 1.35) | 0.811 | 11 | 1.66 (0.80, 3.44) | 0.176 |
| Total monthly distance at > 16.8 m/s - stayers (>2100m) (km) |  | |  |  | 64 | 1.07 (1.01, 1.14) | 0.028 | 64 | 0.82 (0.54, 1.23) | 0.335 |
| Total monthly distance at > 16.8 m/s - stayers (>2100m) (Categorical) |  | |  |  |  |  |  |  |  |  |
| 0 km |  | |  |  | 56 | Ref |  | 56 | Ref |  |
| > 0 km |  | |  |  | 8 | 1.43 (1.22, 1.68) | 0.000 | 8 | 0.66 (0.19, 2.28) | 0.511 |
| Total distance galloped per month – Elite horses (km) |  | |  |  | 64 | 1.01 (1.00, 1.02) | 0.145 | 64 | 1.04 (0.97, 1.10) | 0.264 |
| Total distance galloped per month – Elite horses (Categorical) |  | |  |  |  |  |  |  |  |  |
| ≤ 10 km |  | |  |  | 22 | Ref |  | 22 | Ref |  |
| > 10 km, ≤ 17 km |  | |  |  | 31 | 1.15 (0.89, 1.49) | 0.281 | 31 | 3.23 (0.66, 15.65) | 0.146 |
| > 17 km |  | |  |  | 11 | 1.17 (0.83, 1.64) | 0.364 | 11 | 3.09 (0.66, 14.49) | 0.153 |
| Total monthly distance at 13.3-14.3 m/s – Elite horses (km) |  | |  |  | 64 | 1.00 (0.99, 1.02) | 0.664 | 64 | 1.04 (0.97, 1.11) | 0.312 |
| Total monthly distance at 14.4-15.4 m/s - Elite horses (km) |  | |  |  | 64 | 1.05 (1.01, 1.09) | 0.021 | 64 | 1.12 (0.98, 1.29) | 0.094 |
| Total monthly distance at 14.4-15.4 m/s - Elite horses (Categorical) |  | |  |  |  |  |  |  |  |  |
| 0 km |  | |  |  | 62 | Ref |  | 62 | Ref |  |
| > 0 km |  | |  |  | 2 | 1.14 (0.90, 1.44) | 0.284 | 2 | 1.49 (0.88, 2.52) | 0.141 |
| Total monthly distance at 15.5-16.7 m/s - Elite horses (km) |  | |  |  | 64 | 1.01 (0.98, 1.05) | 0.409 | 64 | 1.06 (1.00, 1.13) | 0.047 |
| Total monthly distance at > 16.8 m/s - Elite horses (km) |  | |  |  | 64 | 1.02 (1.00, 1.03) | 0.102 | 64 | 0.91 (0.76, 1.08) | 0.290 |
| Total monthly distance at > 16.8 m/s - Elite horses (Categorical) |  | |  |  |  |  |  |  |  |  |
| 0 km |  | |  |  | 58 | Ref |  | 58 | Ref |  |
| > 0 km |  | |  |  | 6 | 1.30 (1.08, 1.56) | 0.005 | 6 | 1.36 (0.37, 5.05) | 0.643 |
| Average frequency of fastwork per week |  | |  |  | 66 | 1.03 (0.88, 1.20) | 0.731 | 66 | 2.15 (1.08, 4.29) | 0.030 |
| Number of weeks between racestarts |  | |  |  | 65 | 1.28 (1.03, 1.58) | 0.025 | 65 | 2.10 (0.48, 9.28) | 0.326 |
| **Miscellaneous training variables** |  | |  |  |  |  |  |  |  |  |
| Slow work training surface^c^ |  | |  |  |  |  |  |  |  |  |
| Majority work on dirt | 1 | | 0.00 (0.00, 0.00) | 0.000 | 6 | 0.65 (0.37, 1.13) | 0.130 | 6 | 0.00 (0.00, 0.00) | 0.000 |
| Majority work on turf | 1 | | Omitted |  | 1 | 0.77 (0.50, 1.18) | 0.230 | 1 | 0.89 (0.09, 8.47) | 0.916 |
| Majority work on sand | 42 | | 1.27 (0.46, 3.49) | 0.641 | 56 | 0.66 (0.49, 0.90) | 0.008 | 56 | 1.00 (0.42, 2.42) | 0.992 |
| Majority work on synthetic | 9 | | 0.85 (0.32, 2.23) | 0.740 | 9 | 0.83 (0.64, 1.08) | 0.164 | 9 | 1.61 (0.53, 4.87) | 0.400 |
| Fast work training surface^c^ |  | |  |  |  |  |  |  |  |  |
| Majority work on dirt | 4 | | 1.03 (0.14, 7.41) | 0.978 | 5 | 0.54 (0.29, 1.03) | 0.062 | 5 | 1.90 (0.07, 51.0) | 0.702 |
| Majority work on turf | 10 | | 1.08 (0.23, 5.20) | 0.921 | 15 | 0.82 (0.49, 1.38) | 0.463 | 15 | 1.58 (0.23, 10.9) | 0.642 |
| Majority work on sand | 11 | | 0.92 (0.12, 7.28) | 0.938 | 20 | 0.90 (0.47, 1.70) | 0.743 | 20 | 1.13 (0.07, 19.1) | 0.931 |
| Majority work on synthetic | 25 | | 1.04 (0.16, 6.84) | 0.965 | 31 | 0.96 (0.55, 1.70) | 0.895 | 31 | 2.74 (0.21, 36.2) | 0.445 |
| Alternative exercise methods |  | |  |  |  |  |  |  |  |  |
| Does not use alternative exercise methods | 3 | | Ref |  | 7 | Ref |  | 7 | Ref |  |
| Uses alternative exercise methods | 44 | | 1.28 (0.40, 4.12) | 0.684 | 59 | 1.05 (0.61, 1.83) | 0.853 | 59 | 0.46 (0.17, 1.26) | 0.132 |
| Time on walker per day in minutes | 47 | | 1.00 (0.99, 1.02) | 0.385 | 66 | 1.00 (1.00, 1.00) | 0.546 | 66 | 1.00 (0.98, 1.01) | 0.611 |
| Category of trainer |  | |  |  |  |  |  |  |  |  |
| A | 19 | | Ref |  | 19 | Ref |  | 19 | Ref |  |
| General | 20 | | 0.75 (0.37, 1.51) | 0.420 | 24 | 0.94 (0.74, 1.20) | 0.625 | 24 | 0.92 (0.19, 4.37) | 0.913 |
| Restricted | 8 | | 1.71 (0.81, 3.60) | 0.156 | 23 | 1.00 (0.67, 1.50) | 0.996 | 23 | 0.72 (0.07, 7.18) | 0.776 |
| Location of trainer |  | |  |  |  |  |  |  |  |  |
| Metro | 17 | | Ref |  | 19 | Ref |  | 19 | Ref |  |
| Provincial | 24 | | 1.29 (0.67, 2.48) | 0.449 | 36 | 1.06 (0.82, 1.36) | 0.669 | 36 | 0.58 (0.17, 1.92) | 0.370 |
| Country | 6 | | 3.07 (0.56, 16.86) | 0.198 | 11 | 1.02 (0.69, 1.51) | 0.918 | 11 | 0.59 (0.07, 4.76) | 0.619 |
| Standardised versus Specialised training programs |  | |  |  |  |  |  |  |  |  |
| Standardised | 31 | | Ref |  | 41 | Ref |  | 41 | Ref |  |
| Specialised | 16 | | 0.86 (0.38, 1.93) | 0.715 | 25 | 0.79 (0.60, 1.04) | 0.088 | 25 | 1.19 (0.39, 3.68) | 0.762 |
| Galloped, trialled, or raced two-year-olds |  | |  |  |  |  |  |  |  |  |
| Does not gallop, trial, or race two-year-olds | 0 | | - |  | 8 | Ref |  | 8 | 0.00 (0.00, 0.00) | 0.000 |
| Does not race two-year-olds but gallops or trials them | 4 | | Ref |  | 11 | 1.02 (0.46, 2.29) | 0.962 | 11 | 0.67 (0.12, 3.66) | 0.648 |
| Races two-year-olds | 43 | | 0.76 (0.09, 6.34) | 0.797 | 47 | 1.47 (0.72, 3.00) | 0.295 | 47 | Ref |  |
| Number of two-year-olds horses in the stable | 47 | | 1.00 (0.99, 1.01) | 0.754 | 66 | 1.01 (1.01, 1.01) | 0.000 | 66 | 0.99 (0.97, 1.02) | 0.410 |
| Number of mature (≥ three-year-old) horses in the stable | 47 | | 0.99 (0.98, 1.00) | 0.044 | 66 | 1.00 (1.00, 1.01) | 0.000 | 66 | 1.00 (0.98, 1.01) | 0.762 |
| Number of horses in the stable | 47 | | 1.00 (0.99, 1.00) | 0.094 | 66 | 1.00 (1.00, 1.00) | 0.000 | 66 | 1.00 (0.99, 1.01) | 0.596 |

^a^ 19 trainers did not have any two-year-old race starts within the study period and was not included in denominator data

^b^ No two-year-old CMI were recorded during the study period

^c^ Several trainers were included in more than one of these categories
