# Supplementary material for: The association between Thoroughbred racehorse training practices and musculoskeletal injuries in Victoria, Australia
Source: Front Vet Sci. 2023 Oct 24;10:1260554. doi: 10.3389/fvets.2023.1260554 (PMC10628463; doi:10.3389/fvets.2023.1260554)
Supplement: Supplementary file 2 [file Table_2.DOCX]

Supplementary Table 2: Training program glossary from cluster analyses of progressive (pre-trial) and race-fit gallop work programs of Thoroughbred racehorses in Victoria, Australia (adapted from Morrice-West et al., 2020)^1^.

| Two-year-old progressive programs | Detailed description |
| --- | --- |
| 1. Fast and light | Shortest time in gallop speed training and time to trial, with the lowest cumulative gallop distance. |
| 1. Moderate volume | Moderate time undertaking fast training and time to trial, with a moderate overall gallop distance over the program. This group’s weekly slow speed gallops were similar to group (1), and very high-speed gallops (≥ 16.8 m/s) similar to group (3). |
| 1. High volume over extended time periods | Longest time to trial, with the greatest duration of time in gallop training. Highest overall cumulative gallop distance (≥ 13.3 m/s) with a high cumulative volume of slow speed gallop training (13.3 – 14.3 m/s). |

| Three-year-old and above progressive programs |  |
| --- | --- |
| 1. Fast and light | Shortest time in gallop training and time to trial, with the lowest cumulative and weekly average overall distances (≥ 13.3 m/s) |
| 1. Moderate volume | Moderate time undertaking gallop training and time to trial. Moderate overall cumulative gallop distance. This group’s weekly and cumulative program slow and overall speed gallop distance was between groups (1) and (3), but had the highest weekly average distance above 15.5 m/s. |
| 1. High volume with slower speed gallops | Longest time to trial. Highest amount of slow speed gallop training and cumulative fast gallops (≥ 15.5 m/s) resulting in the highest cumulative gallop distance. |

| Three-year-old and above racefit monthly workloads |  |
| --- | --- |
| 1. Low volume | Lowest total gallop (≥ 13.3 m/s) distance per month including lower distance at slow speed galloping and lower faster gallops (≥ 15.5 m/s). |
| 1. Moderate volume | Combined medium volume and medium-volume with greatest high speed workouts from Morrice-West et al., 2020^1^ (Median combined speeds 8000 (IQR 8000, 9600) and 12800 (IQR 11200, 14400) respectively).  Detailed descriptions of individual programs from Morrice-West et al., 2020:  Medium volume – Moderate total distance, with more slow speed gallops compared to the low volume group and marginally more high-speed work.  Medium volume with greatest high-speed work – Second highest total gallop distance, with moderate slow speed gallops and the highest race-speed gallops (≥ 16.8 m/s) |
| 1. High volume | Highest total gallop distance per month. Highest slow speed gallop training. |

1. Morrice-West, A. V., Hitchens, P. L., Walmsley, E. A., Stevenson, M. A. & Whitton, R. C. Training practices, speed and distances undertaken by Thoroughbred racehorses in Victoria, Australia. *Equine Vet. J.* **52**, 273–280 (2020).
