# Supplementary material for: The association between Thoroughbred racehorse training practices and musculoskeletal injuries in Victoria, Australia
Source: Front Vet Sci. 2023 Oct 24;10:1260554. doi: 10.3389/fvets.2023.1260554 (PMC10628463; doi:10.3389/fvets.2023.1260554)
Supplement: Supplementary file 3 [file Table_3.DOCX]

Supplementary Table 3. Comparison between measures of performance for Victorian trainers, 2013 to 2017 stratified by career and previous season (1 August 2016 to 31 July 2017) wins, places, and prizemoney per start assessed univariably against the study factors in the final multivariable MSI models and MSI outcomes. Trainer measures of performance were previously described in detail by Morrice-West et al., 2021^1^. Wins and places were analysed as negative binomial models, career prizemoney per start was log-transformed and analysed via linear regression, last season prizemoney per start was analysed via logistic regression. Only significant (P≤0.05) relationships between performance outcomes and study factors are included. Significant directions of effect are colour coded by injury risk (green = decreased risk, "positive” effect; red = increased risk, "negative” effect) and performance (green = better performance, "positive” effect; red = poorer performance, “negative” effect), either compared to the reference category (ref) in the case of categorical variables, or as a general effect for an increase in the study factor variable for continuous variables.

| **Study factor** | **n** | **MSI outcomes** | | | **Performance outcomes** | | | | | |
| --- | --- | --- | --- | --- | --- | --- | --- | --- | --- | --- |
|  |  | **Two-year-old injuries** | **Mature (≥ Three-year-old) injuries)** | | **Career** | | | **Previous season** | | |
|  |  | **MSI** | **MSI** | **CMI** | **Wins** | **Places** | **Prizemoney** | **Wins** | **Places** | **Prizemoney^2^** |
| **Two-year-old rest practices**3 **(Categorical)** |  |  |  |  |  |  |  |  |  |  |
| Short and less frequent | 7 | negative |  |  |  |  | NS* |  |  | negative |
| Short and more frequent | 19 | Ref |  |  |  |  | Ref |  |  | Ref |
| Long and less frequent | 12 | negative |  |  |  |  | negative |  |  | negative |
| Long and more frequent | 9 | negative |  |  |  |  | NS |  |  | NS |
| **Mature rest practices (Categorical)** |  |  |  |  |  |  |  |  |  |  |
| Short and less frequent | 10 |  | NS |  | NS | NS | NS |  |  | NS |
| Short and more frequent | 28 |  | Ref |  | Ref | Ref | Ref |  |  | Ref |
| Long and less frequent | 20 |  | NS^§^ |  | negative | negative | negative |  |  | negative |
| Long and more frequent | 8 |  | positive |  | NS | NS | NS |  |  | NS |
| **Two-year-old progressive training programs^3^** |  |  |  |  |  |  |  |  |  |  |
| Fast and light | 19 | Ref |  |  |  |  | Ref |  |  |  |
| Moderate volume | 22 | negative |  |  |  |  | NS |  |  |  |
| High volume over extended time periods | 5 | NS |  |  |  |  | positive |  |  |  |
| **Two-year-old racefit total distance galloped per month at 15.5 – 16.7 m/s (km)^4^** | 45 | positive | positive |  |  |  |  |  | Positive |  |
| **Galloped, trialled, or raced two-year-olds** |  |  |  |  |  |  |  |  |  |  |
| Does not gallop, trial, or race two-year-olds | 8 |  | NS | positive | negative | negative | negative | NS | NS | negative |
| Does not race but gallops or trials two-year-olds | 11 |  | positive | positive | negative | negative | negative | negative | negative | NS |
| Races two-year-olds | 47 |  | Ref | Ref | Ref | Ref | Ref | Ref | Ref | Ref |

*NS = Not significant

^§^ Increased risk compared to long and more frequent as well as short and less frequent categories

^1^Morrice-West A V., Hitchens PL, Walmsley EA, Wong ASM, Whitton RC. Association of Thoroughbred Racehorse Workloads and Rest Practices with Trainer Success. Animals. 2021 Nov 1;11(11):3130

^2^Variable binarized to (0) did not win any prizemoney (1) won prizemoney last season.

^3^Relationships between the study factor and performance outcomes were assessed for the 47 trainers who recorded more than one two-year-old race start (study factor was only significantly associated with MSI in the two-year-old injury model).

^4^Relationships between the study factor and performance outcomes were assessed for the 47 trainers who recorded more than one two-year-old race start as well as for all trainers as the study factor was significantly associated with MSI in both the two-year-old and mature injury models. Previous season places was only significantly associated with the study factor when assessed for the 47 trainers with more than one two-year-old race start but not when assessed for all trainers.
